# What the Papers Say

**DOI:** 10.1093/jhps/hnw010

**Published:** 2016-03-25

**Authors:** Ajay Malviya

**Affiliations:** Consultant Orthopaedic Surgeon, Northumbria Healthcare NHS Foundation Trust, Newcastle upon Tyne, NE12 8EW, United Kingdom

The *Journal of Hip Preservation Surgery* (*JHPS*) is not the only place where work in the field of hip preservation may be published. Although our aim is to offer the best of the best, we continue to be fascinated by work that finds it way into journals other than our own. There is much to learn from it, so *JHPS* has selected six recent and topical articles for those who seek a brief summary of what is taking place in our ever-fascinating world of hip preservation. What you see here are the mildly edited abstracts of the original articles, to give them what *JHPS* hopes is a more readable feel. If you are pushed for time, what follows should take you no more than 10 min to read. So here goes …

## WHAT ARE THE RISK FACTORS FOR CONVERSION TO TOTAL HIP REPLACEMENT AFTER HIP ARTHROSCOPIC SURGERY?

In a population-based analysis, Schairer et al [1] from Hospital for Special Surgery, New York, evaluated the conversion rate to total hip replacement (THR) within 2 years of hip arthroscopy and the influence of age, arthritis and obesity on the rate of conversion.

The data were obtained from the State Ambulatory Surgery Databases and State Inpatient Databases for California and Florida from 2005 to 2012, which contain 100% of patient visits. Hip arthroscopy patients were tracked for subsequent primary THR within 2 years. Out-of-state patients and patients with <2-year follow-up were excluded. Multivariate analysis identified risks for subsequent hip replacement after arthroscopy.

The authors identified 7351 patients who underwent hip arthroscopy with 2-year follow-up. The mean age was 43.9 ± 13.7 years, and 58.8% were female patients. Overall, 11.7% of patients underwent THR conversion within 2 years. The conversion rate was lowest in patients aged <40 years (3.0%) and highest in the 60- to 69-year-old group (35.0%) (*P* < 0.001). An increased risk of conversion to THR was noted in older patients and in patients with osteoarthritis or obesity at the time of hip arthroscopy. Patients treated at high-volume hip arthroscopy centers had a lower THR conversion rate than those treated at low-volume centers (15.1% versus 9.7%, *P* < 0.001).

In this retrospective comparative (Level III) study the authors concluded that older patients have a higher rate of conversion to THR, as do patients with osteoarthritis or obesity.

## RADIOLOGICAL MARKERS FOR IDENTIFYING CAM LESIONS—THE CONCEPT OF OMEGA ANGLE?

Portugese and Spanish researchers [2] have looked at the limitations of using α angle in quantifying cam-type pathology, which measures the deformity in a single plane. However, when the deformity overlaps the superior retinaculum, femoral head osteoplasty in this area can jeopardize intra-articular vascular structures. This study proposes a new angular measure of the linear radial extension of cam deformity as a planning tool for bone resection and compares the accuracy of femoral head osteoplasty using open and arthroscopic surgery.

Twenty-five symptomatic patients operated on for Femoroacetabular Impingement (FAI) were included in this study. Radial magnetic resonance imaging was done before and after surgery. Bi-dimensional coordinates of the vascular foramina and radial extension of the deformity (omega angle) were measured. This extension was correlated with the vascular foramina location and α-angle value. Accuracy of resection and hip function was evaluated before and after surgery.

The cam lesion frequently extended posteriorly. No relationship between values of α and ω angles was found. Cam resection was complete in 88% of cases; there was a significant improvement in outcome score after surgery.

This study showed that α angle, measured in one plane, was not a predictor of the radial extension of cam deformity. To achieve a full resection, it was frequently necessary to extend the femoral head osteoplasty over the retinacular area. Pre-operative determination of the ω angle and location of the vascular foramina helped improve cam resection safety and accuracy.

## DOES PERIACETABULAR OSTEOTOMY FOR HIP DYSPLASIA RESTRICT HIP RANGE OF MOTION?

Simulated range of motion (ROM) analysis performed in Osaka University, Japan, [3] attempted to identify the optimal reorientation of the acetabulum for developmental dysplasia (DDH) of the hip. The simulated ROMs of 52 DDHs after rotational acetabular osteotomy (RAO) with several patterns of femoral head coverage and those of 73 normal hips were analysed using computer models reconstructed from computed tomographic images.

After RAO with a lateral center edge angle (LCEA) of 30° and an anterior center edge angle (ACEA) of 55° producing coverage similar to that of normal hips, the maximal flexion and maximal internal rotation at 110° flexion with 20° adduction were significantly smaller than those of the normal group. To achieve ROMs after RAO similar to those of the normal group, an LCEA of 30° with an ACEA of 45°, an LCEA of 25° with an ACEA of 45° to 50° and an LCEA of 20° with an ACEA of 50° could be preferred angles to target, even though they provided smaller coverage than that of normal hips.

The authors concluded that after RAO producing femoral head coverage similar to that of normal hips, the maximal flexion and the maximal internal rotation at 110° flexion with 20° adduction were significantly smaller than those of the normal group.

## RESULTS OF ANTEVERTING PERIACETABULAR OSTEOTOMY FOR ACETABULAR RETROVERSION

While the role of periacetabular osteotomy (PAO) in correcting acetabular retroversion is well described, there is very little published literature on this particular indication of PAO. A retrospective review performed in Mayo Clinic [4] identified a cohort of patients who underwent RPAO for acetabular retroversion in isolation or in the setting of dysplasia (LCEA ≤ 19°). The acetabular retroversion with FAI was diagnosed clinically and radiographically, with a positive crossover and posterior wall signs on pelvic radiographs.

Twenty-three patients (30 hips) met the inclusion criteria: 20 hips with isolated retroversion and 10 hips with retroversion and hip dysplasia. The average age at the time of the procedure was 26 years (range, 13–45 years). The average length of follow-up was 5 years (range, 2–19 years). Harris Hip Score (HHS) and radiographs were evaluated preoperatively and at last follow-up.

The mean preoperative LCEA was 31° (range, 22°–49°) in the isolated retroversion group and 9° (range, –4° to 17°) in the dysplastic group. Postoperatively, the LCEA in the dysplastic group increased to 35° (range, 15°–46°) (*P* = 0.0001). The crossover sign corrected in 55% (11/20) of the isolated 

retroversion group and 80% (8/10) of the dysplastic group. The acetabular index (mean ± SD) improved from 1.3 ± 0.3 to 1.7 ± 0.6 (*P* = 0.0001), indicating improved anteversion. At the latest follow-up, the average HHS in the isolated 

retroversion group increased from 58 (range, 23–77) preoperatively to 93 (range, 68–100) (*P* = 0.0001); the HHS in the dysplastic group improved from 49 (range, 20–74) to 92 (range, 77–100) (*P* < 0.0001). Complication rates were similar in both groups. Excluding hardware removal, additional surgeries were performed in 13% (4/30).

RPAO performed for FAI in the young patient with isolated acetabular retroversion or retroversion in the setting of dysplasia successfully improved clinical and radiographic results at mid- to long-term follow-up.

## IS CAM DEFORMITY A PRIMARY MALFORMATION OR SECONDARY TO A DEGENERATIVE PROCESS OR REPETITIVE IMPINGEMENT?

Morphological deformities of the hip, such as femoroacetabular impingement, may be responsible for up to 80% of hip osteoarthritis. In cam-type FAI, the pathomechanism has been attributed to repeated abnormal contact between the femur and the antero-superior acetabular rim, resulting in cartilage and labrum degeneration. Subchondral bone stiffness likely plays a major role in the process, but little is known of the mechanical properties of the cam deformity. Haider *et al*. [5] from Ottawa, therefore, set out to look at the femoral subchondral bone properties of patients with Cam-type femoroacetabular impingement.

The purpose of this study was to determine tissue modulus and the trabecular micro-architecture of the subchondral bone of the cam deformity of patients undergoing resection surgery as well as comparing these parameters to healthy age-matched controls.

Twelve osteochondral bone biopsies were obtained from symptomatic FAI patients and 10 osteochondral control specimens were harvested from cadaveric femurs. A combination of mechanical testing, micro-computed tomographic and finite element analysis was used to determine tissue modulus, bone volume fraction, trabecular thickness, trabecular and spacing and trabecular number.

The mean tissue modulus of the cam-type FAI deformities (*E* = 5.4 GPa) was significantly higher than normal controls (*E* = 2.75 GPa, *P* = 0.038), but no statistically significant differences were found in bone micro-architectural parameters.

These data suggest that subchondral bone of the cam deformity consists of older secondary mineralized bone. This would support the theory that the cam deformity is a primary malformation with intrinsic biomechanical abnormalities, rather than a secondary deformity as a part of the degenerative process of the covering cartilage or remodeling due to repeated impingement.

## STRATEGIES TO PREVENT HETEROTOPIC OSSIFICATION AFTER HIP ARTHROSCOPY: ROLE OF NAPROXEN

In a double-blind randomized control trial Beckmann et al [6] sought to determine the effect of postoperative naproxen therapy on the development of heterotopic ossification (HO) following arthroscopic surgery for femoroacetabular impingement.

Between August 2011 and April 2013, 108 eligible patients were enrolled and randomized to take naproxen or a placebo for 3 weeks postoperatively. Radiographs were made at routine follow-up visits for 1 year following surgery. The primary outcome measure was the development of HO, as classified with the Brooker criteria and two-dimensional measurements on radiographs made at least 75 days postoperatively (average, 322 days). The primary analysis, performed with a Fisher exact test, compared the proportion of subjects with HO between the treatment and control groups. A single *a priori* interim analysis was planned at the midpoint of the study.

The local safety and monitoring board stopped this study when the interim analysis showed that the stopping criterion had been met for demonstration of efficacy of the naproxen intervention. The prevalence of HO was 46% (22 of the 48 in the final analysis) in the placebo group versus 4% (2 of 48) in the naproxen group (*P* < 0.001). Medication compliance was 69% overall, but it did not differ between the naproxen and the placebo groups. Minor adverse reactions to the study medications were reported in 42% of the patients taking naproxen versus 35% of those taking the placebo (*P* = 0.45).

The authors therefore concluded that prophylaxis with naproxen was effective in reducing the prevalence of HO without medication-related morbidity. 

## CONFLICT OF INTEREST STATEMENT

None declared. 
